# 老年滤泡性淋巴瘤诊断与治疗中国专家共识（2024年版）

**DOI:** 10.3760/cma.j.cn121090-20240701-00243

**Published:** 2024-09

**Authors:** 中国抗癌协会血液肿瘤专家委员会 中华医学会血液学分会淋巴细胞疾病学组 中国滤泡淋巴瘤工作组 中国老年医学学会血液学分会 中国抗癌协会淋巴瘤专家委员会 中国老年保健协会淋巴瘤专家委员会

## Abstract

随着人口老龄化来临，老年滤泡性淋巴瘤（FL）患者比例上升。老年FL患者的治疗强调个体化原则，以改善肿瘤相关症状为前提，生活质量的改善为目标。因此中国抗癌协会血液肿瘤专业委员会、中华医学会血液学分会淋巴细胞疾病学组、中国滤泡淋巴瘤工作组、中国老年医学学会血液学分会、中国抗癌协会淋巴瘤专业委员会和中国老年保健协会淋巴瘤专业委员会组织相关专家制订了本共识。

## Abstract (en)

In the general population of China, the number of older patients with follicular lymphoma（FL）will continue to increase with age. The treatment of elderly patients with FL is based on individual principles, which focuses on the improvement of tumor-related symptoms and quality of life. To improve the diagnosis and treatment of elderly patients with FL in China, several organizations（i.e., Hematology Oncology Committee of China Anti-Cancer Association, etc.）have gathered relevant experts to formulate this consensus.

滤泡性淋巴瘤（FL）是一类起源于滤泡生发中心B细胞、形态上保留了部分滤泡结构的非霍奇金淋巴瘤（NHL），约占B细胞NHL的8％～23％，中位诊断年龄约为53岁[1]，发病率从35岁开始逐步增加，至70岁达到峰值。年龄是影响FL患者预后的因素之一[2]，随年龄增长患者的生存概率呈阶梯式下降[3]–[4]。国内一项单中心回顾研究报道60岁及以上FL患者5年总生存（OS）率仅为46.5％[5]。尽管疾病本身依然是FL患者死亡的重要原因[6]，但在老年患者中淋巴瘤导致患者死亡比例仅占30％左右[7]。相反，因自身合并症增多[8]，70岁以上患者起始治疗1年内非复发死亡风险高达14％[9]。我国正面临快速的人口老龄化[10]，老年FL患者未来占比将会逐渐增加，亟需重点关注。

FL呈惰性且绝大多数不可治愈，其生物学及临床特征异质性极强，治疗手段多样化。多数老年FL患者因合并症等原因被临床研究排除[11]，而真实世界中老年FL患者治疗相关的死亡风险比较高，因此老年FL患者诊断及治疗无标准化流程遵循，更强调个体化治疗原则。为提高我国临床医师对老年FL的诊治水平，结合国际相关指南[12]制定了本共识。

一、老年患者的定义

年龄大于60岁是FL患者不良预后因素，因此，本共识将年龄≥60岁认定为老年FL患者临界值。60岁以上人群慢性病共病患病率为43.6％[8]，造成不同程度的功能丧失，导致治疗相关死亡风险增加，如何评估其治疗耐受性显得尤为突出。目前尚无通过评估老年FL患者耐受性进行治疗决策的相关研究。老年综合评估（CGA）联合多学科会诊深入且全面客观评估老年FL患者治疗选择和耐受性，可辅助指导治疗决策的制定。本共识建议参照北京医院的临床研究[13]，具体评分细则和分组依据见表1、2。所谓适合化疗者，是以能耐受标准剂量R-CHOP（利妥昔单抗+环磷酰胺+阿霉素+长春新碱+泼尼松）方案为依据。

**表1:** 老年综合评估量表

量表	评分内容
ADL	洗澡、穿衣、如厕、移动、进食、大小便控制能力
IADL	独立使用电话、购物、做饭、家务管理、洗衣服、出行、服药、理财
MCIRS-G	根据循环系统、消化系统、肾脏系统等14个系统损害程度进行评分： 0分：没有损害； 1分：轻度损害但不干扰正常生活，无需治疗，预后良好； 2分：中度损害，干扰正常生活，需要治疗，预后良好； 3分：重度损害，可能致残，需要立即治疗，预后较差； 4分：极重度损害，需要紧急治疗，预后严重

**注** ADL：日常生活活动功能评定量表；IADL：工具性日常生活活动功能评估量表；MCIRS-G：改良老年疾病累计评分表

**表2:** 老年综合评估评分标准

组别	ADL（分）	IADL（分）	MCIRS-G	年龄
适合化疗^a^	6	8	无3～4级合并症（且2级合并症<5个）	<80岁
不适合化疗^b^	5	6～7	无3～4级合并症（且2级合并症5～8个）	≥80岁适合化疗患者
脆弱^b^	<5	<6	≥1个3～4级合并症（或2级合并症>8个）	≥80岁不适合化疗患者

**注** ADL：日常生活活动功能评定量表；IADL：工具性日常生活活动功能评估量表；MCIRS-G：改良老年疾病累计评分表；^a^表示均需符合；^b^表示符合1项即可

二、临床表现

多数患者因其他原因就诊无意发现而诊断，绝大部分以颈部或腹部无痛性淋巴结肿大为首要表现，进展期可出现结外淋巴组织、器官或淋巴系统外部位侵犯，产生因肿块压迫的局部症状或浸润导致的功能缺失。淋巴组织以外部位为首发表现的相对较少，以皮肤、睾丸、卵巢等报道较多，预后相对较好。约80％的患者在诊断时已处于晚期。出现B症状的患者比例不足20％，美国东部肿瘤协作组（ECOG）体能评分≥2分、HGB≤120 g/L及LDH和β_2_微球蛋白水平升高等特征在老年FL患者中出现比例较高[14]。

三、发病机制

FL发病机制大致可分为3个阶段[15]–[18]：B细胞在骨髓发育至前体B细胞阶段，位于14q32的IGH基因与位于18q21的BCL2基因融合形成t（14;18）（q32;q21），导致BCL2蛋白过表达，B细胞因此获得抗凋亡能力，携带t（14;18）重排的B细胞称之为FL样细胞，在骨髓发育成熟后进入外周淋巴结初级淋巴滤泡；滤泡内B细胞组成性表达活化诱导的胞苷脱氨酶，保留了体细胞高频突变和类别转换的能力，同时其表面BCR受体可变区域进行N-糖基化修饰，与滤泡内被巨噬细胞等提呈的内源性凝集素结合，从而获得自身生存优势及持续增殖的能力，并定植于滤泡生发中心内，为原位滤泡B细胞肿瘤（ISFN），部分成为记忆B细胞；携带上述特征的记忆B细胞在次级淋巴滤泡内反复循环，进一步造成其基因组不稳定，出现额外的基因突变（大多为表观遗传调控相关基因），最终促使FL发生。此外，肿瘤微环境内部细胞成分及其表型、功能等均不同，且与FL细胞存在持续、动态的交互作用，由此形成个体之间、个体不同部位及病程不同阶段等的差异，是临床异质性的基础。

约20％ FL患者无t（14;18）（q32;q21），但基因突变与拷贝数变异与经典型FL（cFL）相似，仅突变发生频率不同，如STAT6（57％）、CREBBP（49％）突变频率较高，而KMT2D突变频率较低（27％）[19]，STAT6的突变激活在无IGH::BCL2的情况下可能提供了新的抗凋亡机制[20]。

尽管目前尚无关于老年FL的生物学特征系统研究，但老年患者淋巴结的结构、细胞成分及功能均有其自身的特点，如纤维网状细胞、滤泡树突状细胞等不仅表现为数量减少，而且分泌功能下降，免疫细胞呈现衰老状态等[18]，因此推测老年FL可能具有独特的生物学特点。已有的研究显示FL患者基因突变数量随年龄增长而上升，但多为同义突变或错义突变，对疾病进程和演化的影响较小[9]。

四、病理诊断

取材要点、组织形态及免疫表型等参考《中国滤泡性淋巴瘤诊断与治疗指南（2023年版）》[21]。

以下情况可考虑分子病理学检查：①用于鉴别诊断，如滤泡性大B细胞淋巴瘤（即第4版世界卫生组织分类的FL-3B级）年轻患者表达IRF4/MUM1时，需评估是否存在IRF4基因重排；组织学表现以弥漫性生长方式为主、临床表现为腹股沟区域大包块的患者，建议进行BCL2重排、STAT6突变及1p36缺失或TNFRSF14突变等检测，以协助判断是否为具有显著弥漫生长模式的FL。②拟行临床生物学模型进行预后判断，如m7-FLIPI评分等。③指导靶向药物治疗，如他泽司他治疗前建议行EZH2突变检测。④其他。

2022年世界卫生组织和国际临床咨询委员会分别对成熟B细胞肿瘤进行了更新[22]–[23]，新分类均更加注重疾病发病机制对于疾病界定的影响，区别在于对无BCL2重排疾病的归类。表3列出两个机构的FL分类。

**表3:** 世界卫生组织（WHO）和国际临床咨询委员会（ICC）对滤泡性淋巴瘤（FL）分类更新比较

《WHO造血淋巴肿瘤分类》第5版	ICC 2022年版
原位滤泡B细胞肿瘤	FL
FL	原位滤泡性瘤变
经典型FL	十二指肠型FL
滤泡性大B细胞淋巴瘤（FLBCL）	BCL2重排阴性，CD23阳性滤泡中心淋巴瘤
具有不常见特征FL[具有不常见细胞学特征（中等大小母细胞样、大中心细胞样）型和（或）显著弥漫生长模式]	大B细胞淋巴瘤伴IRF4重排
	睾丸FL
儿童型FL	儿童型FL
十二指肠型FL	原发性皮肤滤泡中心淋巴瘤

五、检查、分期及预后评估

病史采集需重点关注既往病史、合并用药等。必要的检查包括：①体格检查：需重点关注浅表淋巴结和肝、脾是否肿大，参考ECOG标准进行体能状况评分；②实验室检查：包括全血细胞、血生化、血清LDH、β_2_微球蛋白、免疫球蛋白定量以及乙型肝炎筛查；③影像学检查：优先推荐PET-CT检查，次选颈、胸、腹、盆腔增强CT检查（如果明确分期为Ⅲ～Ⅳ期患者，以上检查可推迟至启动治疗时）；局限期患者拟采取单纯局部放疗或临床出现不明原因血细胞减少时推荐行骨髓活检+细胞形态学检查+流式细胞术检测，其中骨髓活检样本长度应该在1.5 cm以上；采取含蒽环类药物化疗前，推荐行心脏彩超+射血分数检查。

临床分期用于评估肿瘤侵犯范围，目前采用的是2014版Lugano分期标准[24]，具体见表4。本分期不适用于原发性皮肤滤泡中心淋巴瘤等少见类型。

**表4:** 2014版Lugano分期标准

分期	侵犯范围
局限期	
Ⅰ期	仅侵及单一淋巴结区域（Ⅰ期），或仅单一结外病变不伴淋巴结受累（ⅠE期）
Ⅱ期	侵及横膈一侧≥2个淋巴结区域（Ⅱ期），或伴同侧淋巴结引流区域的局限性结外部位受累（ⅡE期）
进展期	
Ⅲ期	侵及横膈上下淋巴结区域，或横膈以上淋巴结区域受侵伴脾脏累及
Ⅳ期	侵及淋巴结引流区域外非连续部位

**注** 扁桃体、韦氏环和脾脏属于结内组织或器官

六、危险分层

目前尚无专门针对老年FL患者的预后评估模型，仍采用临床广泛使用的FL国际预后指数（FLIPI），即FLIPI-1和FLIPI-2。FLIPI-1指标包括：年龄≥60岁；Ann Arbor分期Ⅲ～Ⅳ期；HGB<120 g/L；血清LDH升高；受侵淋巴结区≥5个。每项计1分，0～1分为低危组，2分为中危组，≥3分为高危组。将包括无治疗指征在内的患者分为低危、中危和高危组，10年OS率分别为71％、51％和36％。FLIP1-2为仅对接受治疗患者的预后评估，指标包括：年龄≥60岁；淋巴结长径>6 cm；骨髓侵犯；β_2_微球蛋白升高；HGB<120 g/L。每项计1分，0分为低危组，1～2分为中危组，≥3分为高危组。低危、中危和高危患者的5年无进展生存（PFS）率分别为79％、51％和20％；对接受含利妥昔单抗（R）的免疫化疗患者，5年PFS率分别为98％、88％和77％。老年FL患者中高危组较多，疾病相关死亡风险较年轻患者显著升高[6]。

七、一线治疗

本共识的治疗推荐主要针对经CGA判定为不适合化疗和脆弱的老年患者，对经CGA判定为适合化疗的患者治疗则参考《中国滤泡性淋巴瘤诊断与治疗指南（2023年版）》[21]。由于相关临床研究较少，治疗推荐并非基于临床研究入排标准，而是根据临床疗效及安全性，结合老年患者虚弱原因等个体情况给出。总体原则是在具有治疗指征的患者中，进一步评估疾病进展风险、宿主预期寿命和安全性等问题，治疗在疗效与安全性和改善生活质量与延长寿命之间作出平衡。R治疗时代，FL患者接受一线、二线及三线治疗后中位PFS期分别为4.8年、1.6年和1年[25]，可作为决策参考。

（一）局限期治疗

Ⅰ～Ⅱ期老年患者同样应以积极治疗为主，期望得到根治为目的。局部放疗并未增加老年患者治疗相关不良反应（AE）[26]。受累部位放疗（ISRT）是Ⅰ期和连续Ⅱ期患者的标准治疗，推荐放疗剂量为24 Gy，分12次。国际淋巴瘤放射肿瘤学组的一项回顾性研究[27]显示，Ⅰ期、Ⅱ期患者接受ISRT后，5年疾病无进展（FFP）率分别为74.1％和49.1％。局部放疗联合CD20单抗可进一步延长PFS期[28]，从而降低部分老年患者接受二线治疗的概率，可作为治疗选择。一些特殊部位（如眼眶等），考虑到放疗相关AE，推荐放疗剂量为4 Gy，分2次。对于不适合放疗的特殊部位（如腹膜后或肠系膜淋巴结）的老年FL患者，可考虑CD20单抗每周1次，共4次治疗，剂量为375 mg/m^2^。早期一项回顾性研究显示[29]，局限期患者采取等待观察策略，63％患者在中位随访86个月时仍未启动抗淋巴瘤治疗。因此，对于完全手术切除的Ⅰ期[30]和不耐受ISRT AE的Ⅰ～Ⅱ期患者，也可选择等待观察[28],[31]。

对于非连续性Ⅱ期的治疗可结合患者预期寿命等具体情况作出选择：①CD20单抗每周1次，共4次治疗，剂量为375 mg/m^2^；治疗后未获得完全缓解（CR）的患者可予以局部放疗。②按照进展期患者管理。

（二）进展期治疗

进展期患者的治疗以缓解症状、延缓疾病进展及改善生活质量为目的。因此治疗前需评估是否具有治疗指征，具体参考表5。重点说明：指标仅作参考，症状是关键，更强调动态变化，这点在老年FL患者治疗决策中尤为重要。比如数年缓慢增大达7 cm及以上的包块，如果无症状，仍可观察。

**表5:** Ⅲ～Ⅳ期滤泡性淋巴瘤患者的治疗指征

治疗指征	临床表现
B症状或皮肤瘙痒	B症状包括：38 °C以上不明原因发热、盗汗、6个月内体重降低>10％
异常体征	出现脾脏肿大、胸腔积液、腹水等
重要器官受损	重要器官受累，导致器官功能损害
血液指标	肿瘤相关的血细胞减少（WBC<3.0×10^9^/L、HGB<100 g/L、PLT<100×10^9^/L）；白血病表现（肿瘤细胞>5.0×10^9^/L）
巨大肿块	淋巴结区累及数量≥3个，长径均≥3 cm或任何一个淋巴结或结外肿块长径≥7 cm
持续肿瘤进展	2～3个月内肿块增大20％～30％，6个月内肿块增大约50％

1. 无治疗指征患者的管理

对于无治疗指征的患者，启动抗淋巴瘤治疗的中位时间为31个月[32]–[33]，目前优先推荐采取观察等待。早期多项Ⅲ期对照研究均提示R短程治疗（每周1次，共4次）尽管并未改善无治疗指征患者的OS期，但会显著延长疾病进展时间和启动下一次抗淋巴瘤治疗时间（TTNALT），中位TTNALT约9.9年[32]–[34]。预期寿命不足10年的患者如果诊断时即采取R短程治疗，可能终身都无需进一步治疗。多项回顾研究提示患者诊断时具有结外或多个淋巴结部位累及、PET-CT提示肿瘤代谢体积较高或LDH快速升高、FLIPI评分高危等特征，预示疾病可能会在较短时间内进展[35]–[39]。因此识别可能会出现短期进展的患者，也可给予早期干预。

2. 有治疗指征患者的管理

不适合化疗和脆弱老年患者的治疗应该以延长TTNALT为主，兼顾安全性及改善生活质量的原则。根据治疗前PET-CT代谢活性指导化疗方案的选择的证据目前尚不足。

GALLIUM研究对比了奥妥珠单抗联合化疗（G-化疗组）和R联合化疗（R-化疗组）在初治FL中的疗效，获得部分缓解以上疗效患者继续接受奥妥珠单抗和R维持治疗2年或至疾病进展，70岁以上或具有合并症的患者各占约20％，7年OS率分别为88.5％和87.2％，3年未启动新抗淋巴瘤治疗（NALT）比例分别为87％和81％，7年时仍未启动NALT比例分别为74.1％和65.4％；在G-化疗组中，化疗方案选择苯达莫司汀、CHOP或CVP（环磷酰胺+长春新碱+泼尼松）方案，3年未启动NALT比例均为87％；联合苯达莫司汀组发生感染的风险增高，尤其是老年患者，而联合CHOP组骨髓抑制发生比例较高[40]–[41]。基于以上，本共识建议奥妥珠单抗或R联合CVP方案作为优先选择。

PRIMA研究[42]显示，R-CHOP/CVP方案诱导治疗后，给予2年R维持治疗（375 mg/m^2^，每2个月1次）可显著延长FL患者的PFS期，但中位随访9.8年时，观察组仍有44.6％的患者未接受下一次抗淋巴瘤治疗，中位TTNALT为6.1年，且因疾病进展导致的死亡风险并未增加。国内回顾性研究显示，依据FLIPI评分危险度进行分层维持治疗，但无一致的结论[43]–[44]。因此，对于不适合化疗或超高龄患者，根据患者情况也可选择观察策略。

RELEVANCE研究[45]评估R联合来那度胺（R2）或化疗（CHOP、苯达莫司汀、CVP分别占72％、23％和5％）对初治FL患者的疗效，6年PFS率分别为60％和59％，且两组在TTNALT、二线治疗的疗效及转化发生概率等方面的差异均无统计学意义；亚组分析显示老年患者同样可获益于R2方案治疗，但安全性方面两组存在区别，联合化疗方案以3～4级（发热性）中性粒细胞减少为多见，而联合来那度胺方案的3～4级皮肤反应更多。GALEN研究[46]评估G联合来那度胺（GL）方案在初诊FL患者中的疗效及安全性，入组100例患者，中位年龄60.5（32～89）岁，70岁以上占17％；诱导治疗结束时总反应率为94％，CR率为80％；中位随访3.7年时，预估3年PFS率为82％，仍未启动NALT患者比例为87％；引起治疗中断的常见原因分别为中性粒细胞减少（37％）、感染（20％）、输液相关反应（17％）和皮肤反应（14％）。基于这两项研究，本共识建议对于肾功能良好且既往无血栓病史或无血栓高危因素的老年患者，在抗血栓预防基础上，R2或GL方案可作为一线治疗选择。

SAKK35/98研究[47]–[48]比较了R短疗程和长疗程（每周1次，共4次；再每2个月1次，共4次）巩固方案对FL的疗效，既往未经治疗的患者在12周的反应率为67％；随访8年，接受长疗程患者中约45％仍保持无事件生存，而未接受继续治疗者为22％。因此，对于临床无紧急降低肿瘤负荷情形的老年患者，R短疗程诱导治疗获得反应后予以R长疗程维持治疗策略也可作为选择。

八、难治/复发患者的治疗

原发难治老年FL患者的治疗较为棘手，可考虑EZH2抑制剂、双特异性抗体及CAR-T细胞治疗等，但真实世界数据有限。复发的老年FL患者仍有相当比例无需进一步治疗。复发时的分期、肿瘤负荷、是否发生转化、既往治疗效果及安全性、宿主CGA评分等因素均影响治疗决策。复发时需要动态评估宿主CGA评分，另外难治/复发老年FL患者建议再行PET-CT检查评估疾病分期、肿瘤负荷等，同时可指导高代谢部位活检，明确是否发生转化[49]。

一项开放性、单臂、Ⅱ期临床研究[50]评估他泽司他（tazemetostat）在二线以上难治/复发FL患者（EZH2野生型为54例，EZH2突变型为45例）中的疗效，结果显示他泽司他单药治疗EZH2突变型和EZH2野生型患者的有效率（ORR）分别为69％和35％；中位PFS期分别为14个月和11个月。

ROSEWOOD研究[51]对比了ZO（泽布替尼+奥妥珠单抗）方案与奥妥珠单抗单药在难治/复发FL患者中的疗效和安全性，ZO组患者的中位年龄是63岁，ORR 69％，CR率39％，中位PFS期达到28.0个月。在≥65岁患者中ZO组ORR为68％，≥75岁患者中ZO组的ORR为73％。在总人群中ZO组ICR评估的安全性方面，ZO组房颤和高血压的发生率较低（均为3％），≥3级中性粒细胞减少和贫血发生率分别为24％和5％。

双特异性单抗对难治/复发FL疗效满意，GO29781研究[52]莫妥珠单抗单药治疗重度难治/复发FL患者，入组患者中位年龄为60（29～90）岁，ORR 78.9％，CR率57.8％，中位PFS期为17.9个月。主要AE为细胞因子释放综合征，发生比例为44.4％，但多为1～2级。

ZUMA-5临床试验[53]入组了中位年龄为60岁的患者，结果显示，既往已接受过2种及以上治疗复发的FL患者接受单次CAR-T细胞治疗的ORR 94.0％，CR率79.0％，中位缓解持续时间（DOR）为38.6个月，中位PFS期为40.2个月，3年时仍未启动NALT比例为60.0％。整体安全性可控。

针对经CGA判定为不适合化疗和脆弱的难治/复发老年FL患者，可供选择的治疗非常有限。治疗选择更多依赖经济、药物可及性及家庭支持等。对于前线治疗1年以上复发的部分患者，R或奥妥珠单抗单药治疗仍作为优先推荐[47],[54]。基于他泽司他安全性特点，其可作为部分不适合化疗和脆弱的难治/复发老年FL患者的二线治疗选择。双特异性抗体及CAR-T细胞治疗等治疗方法在不适合化疗和脆弱的难治/复发老年FL患者中的应用的数据有限，有经验的中心可考虑。

九、疗效评价及随访

参考2014年Lugano会议修订标准评价疗效[24]。老年FL患者疗效更关注症状控制，非CR；此外目前广泛使用的无化疗方案，其有效性评估是30个月时的CR，因此在疾病无进展或耐受许可下，无需频繁评估肿瘤疗效，应密切监测治疗相关并发症。
